# Game-theoretic modeling in regulating greenhouse gas emissions

**DOI:** 10.1016/j.heliyon.2024.e30549

**Published:** 2024-04-30

**Authors:** Oleksandr Maevsky, Maya Kovalchuk, Yuri Brodsky, Valentyna Stanytsina, Volodymyr Artemchuk

**Affiliations:** a Zhytomyr Polytechnic State University, Zhytomyr, Ukraine; b Polissia National University, Zhytomyr, Ukraine; c General Energy Institute of NAS of Ukraine, Kyiv, Ukraine; d National University of Life and Environmental Sciences of Ukraine, Kyiv, Ukraine; e State Institution “Center for Evaluation of Activity of Research Institutions and Scientific Support of Regional Development of Ukraine of NAS of Ukraine”, Kyiv, Ukraine; f G.E. Pukhov Institute for Modelling in Energy Engineering of the NAS of Ukraine, Kyiv, Ukraine; g Center for Information-analytical and Technical Support of Nuclear Power Facilities Monitoring of the NAS of Ukraine, Kyiv, Ukraine; h Kyiv National Economic University Named After Vadym Hetman, Kyiv, Ukraine; i National Aviation University, Kyiv, Ukraine

**Keywords:** Greenhouse gas emissions, Game-theoretic modeling, Environmental policy, Differential equations, Technogenic impact, Emission regulation, Systems analysis, AI in environmental management, Mathematical modeling in ecology

## Abstract

This research introduces an innovative framework for addressing the escalating issue of greenhouse gas emissions through the integration of game theory with differential equations, proposing a novel model to simulate the regulatory dynamics between emission sources and legislative actions. By blending advanced mathematical modeling with environmental science, this paper underscores the critical necessity for pioneering, proactive strategies in environmental management and policy formulation. Central to our approach is the simulation of interactions within a game-theoretic context, aiming to delineate optimal strategies for emission sources and regulatory bodies, factoring in legislative constraints and environmental ramifications. The methodology employs a system of ordinary differential equations, capturing the dynamic, non-stationary nature of atmospheric processes and offering a realistic portrayal of the challenges in mitigating greenhouse gas emissions. Furthermore, the study introduces a fee-based regulatory mechanism designed to encourage emission reductions, highlighting the economic implications of such strategies. Significantly contributing to environmental management, this research presents a detailed model capable of predicting the trajectory of greenhouse gas emissions over a decade, considering the potential impact of technological innovations in emission control. The conclusion emphasizes the promising role of artificial intelligence in refining environmental governance, acknowledging the complexities and limitations inherent in predictive modeling. Aimed at policymakers and environmental scientists, this paper serves as a strategic tool for informed decision-making, advocating for a multidisciplinary approach to develop sustainable, effective solutions to combat one of the most critical environmental challenges facing the globe today.

## 1 Introduction

### 1.1 Technogenic challenges and environmental sustainability

In today's context, the preservation of the ecological state of the environment emerges as a particularly pertinent issue, serving as a fundamental factor in the planned development of humanity. Society and the environment are two integral components of the overall planetary system, exhibiting a clearly defined reciprocal influence. Currently, it is crucial to understand and develop new concepts, approaches, and methods for the timely identification, analysis, and decision-making regarding not just local but primarily global crisis processes (situations, phenomena). A key aspect of this endeavor, especially for the scientific community and all progressive forces of humanity, is the analysis of systemic mechanisms that lead to crises and the development of corresponding models, algorithms, and technologies. These tools are vital not only for crisis mitigation but, more importantly, for the possibility of its prevention. Therefore, a significant contemporary challenge (problem) is to study (research) the general mechanisms of global risks to acquire new reliable knowledge, develop new theories, and make adequate systemic decisions. Partial, local decisions within the scope of monitoring crisis phenomena and anthropogenic impact, etc., are insufficient to address the problem of not only timely prevention but also the halting of the development of both local and global catastrophes.

Given the technogenic specificity of societal development, the environment is subjected to anthropogenic load, predominantly of a negative nature. For instance, sources of greenhouse gas emissions, by creating the well-known ‘greenhouse effect’, contribute to the rapid deterioration of the environmental state. Considering the impossibility of fully neutralizing the negative impact of anthropogenic factors on the environment, the authors propose an information technology for decision-making regarding the optimization of technogenic impact on the environment. This technology particularly focuses on regional sources of greenhouse gas emissions by predicting their concentration using various modern technologies to reduce the volume or concentration of emissions, and accordingly, to regulate responsibilities within the existing legal framework regarding atmospheric emissions of pollutants.

Thus, in such extensive interdisciplinary research, the role is not so much in creating separate mathematical models as in constructing a simulation system. This system should provide tools for analysis, assessment, forecasting, and preparation of adequate managerial decisions. Some components of this system were proposed in publications [[1], [2], [3]]. Based on the above, the functioning of greenhouse gas emission sources and the policy of regulating responsibilities according to current legislation on atmospheric pollutant emissions can be viewed as participants in a ‘game system’. In this scenario, the optimal strategy for the ‘emission source’ player involves staying within established limits regarding concentration or volume of emissions where the ‘penalty’ from the ‘active legislation’ player is minimal.

### 1.2 Literature review

The burgeoning global concern over greenhouse gas emissions and their far-reaching implications underscores the need for a multi-faceted exploration of this environmental challenge. A plethora of scholarly works delve into various aspects of greenhouse gas emissions, spanning from their fundamental environmental impacts to the intricate intricacies of modeling, policy formulation, and comprehensive resolution strategies.

Central to this discourse is the body of literature addressing the overarching problem of greenhouse gas emissions, their origins, impacts, and the critical necessity for efficacious management tactics. Mora et al. [4] shed light on the profound impact of climate change on human pathogenic diseases, illustrating that a significant proportion of infectious diseases are aggravated by climatic hazards. This finding underscores the critical need for reducing greenhouse gas emissions as a means to mitigate extensive health risks. In a similar vein, Sovacool et al. [5] present a critical review of the environmental impacts and policy options related to industrial F-gases, known for their potent greenhouse effects and rising emission rates. Their research emphasizes the urgency of devising comprehensive strategies to counteract these 'super pollutants' and ensure alignment with global climate agreements. Exploring renewable energy as a key player in the race to achieve carbon neutrality, Yuan et al. [6] demonstrate that augmenting renewable energy consumption markedly reduces CO_2_ emissions, thereby underscoring its pivotal role in global emission reduction efforts. Complementing this perspective, Rennert et al. [7] highlight the often-underestimated social cost of carbon dioxide, advocating for refined estimation methods that significantly elevate its assessed value. This revelation accentuates the enhanced benefits of greenhouse gas mitigation and the imperative for more rigorous climate policies. The interplay between climate change, fossil fuel pollution, and children's health is intricately examined by Perera and Nadeau [8], who pinpoint the heightened vulnerability of younger populations to these environmental threats. Their study advocates for urgent measures to shield children's health from the adverse effects of air pollution and climate change. In a study focused on China, Ali et al. [9] uncover the long-term positive impact of renewable energy on reducing emission intensity, thereby endorsing ongoing investments in renewable energy and clean energy innovations. Osman et al. [10] review the cost, environmental impact, and resilience of renewable energy in the face of climate change. Their work highlights the significant potential of renewable sources in combatting global warming, despite the challenges posed by climatic shifts. Hanna et al. [11] propose the emergency deployment of direct air capture technology as a response to the climate crisis, underscoring the substantial yet limited potential of this technology in achieving temperature targets. They call for immediate investment in carbon removal technologies for effective climate change mitigation. In the context of the European Union, Tutak and Brodny [12] assess the impact of renewable energy consumption across various economic sectors, observing its positive influence on economic growth and greenhouse gas reduction. Their findings reveal the diverse effectiveness of renewable energy utilization among EU member states. Terlouw et al. [13] present a life cycle assessment of direct air carbon capture and storage, showcasing its potential for negative greenhouse gas emissions and the significance of geographical location and energy sources in the efficacy of these technologies. Lastly, Buberger et al. [14] conduct an exhaustive analysis of the total life-cycle CO_2_-equivalent emissions from a range of passenger cars. Their study advocates for a transition to electric and hybrid vehicles as a critical step in diminishing the climate impact of the transportation sector.

The exploration of various modeling techniques in the field of greenhouse gas emissions is pivotal for understanding and predicting emission patterns. These studies offer insights into the effectiveness of different predictive models and their applicability in diverse contexts. Bakır et al. [15] focus on forecasting India's future greenhouse gas trajectory using metaheuristic algorithms, highlighting the significant increase in CO_2_ and F-gases emissions by 2050. Their research underscores the necessity for policymakers to take proactive steps in mitigating these emissions and revising energy investments. Adame et al. [16] integrate global datasets to model mangrove carbon emissions, projecting significant emissions by the end of the century if current rates of mangrove loss continue. This study emphasizes the critical need for policies to manage emissions from mangrove loss and the drivers behind it. Gao et al. [17] propose a novel fractional grey Riccati model for CO_2_ emission prediction, combining the Environmental Kuznets Curve hypothesis with differential information principles. Their model demonstrates superior estimation and short-term carbon emission forecasting, highlighting its effectiveness in the United States, China, and Japan. Qader et al. [18] apply multiple methods like neural networks and Gaussian Process Regression for forecasting CO_2_ emissions due to electricity generation in Bahrain. Their research indicates the neural network model's superior performance in forecasting CO_2_ emissions, suggesting its potential application in other regions. Kazancoglu et al. [19] use grey prediction to estimate greenhouse gas emissions from road transport in four European countries, aiming to provide a basis for sustainable development and reduction strategies in road transportation. Nematchoua et al. [20] evaluate and compare carbon emissions and energy requirements in urban, rural, and sustainable neighborhoods, offering recommendations for reducing emissions and achieving ‘zero carbon’ objectives by 2050. Saha et al. [21] demonstrate the effectiveness of machine learning in predicting agricultural nitrous oxide emissions, significantly improving field-level flux predictions when coupled with a cropping systems model. Their approach offers a promising avenue for better predictions of agricultural N_2_O emissions and more effective mitigation strategies. Hassan et al. [22] develop a chaotic artificial ecosystem-based optimization algorithm for the Combined Economic Emission Dispatch problem, aiming to achieve economical operation of electrical power systems while reducing environmental pollution. Their results showcase the algorithm's superiority in finding optimal solutions to reduce emissions in different power generation scenarios. Finally, Bokde et al. [23] propose short-term CO_2_ emissions forecasting methods to enable intelligent scheduling of flexible electricity consumption, thereby minimizing CO_2_ emissions. Their novel forecasting method provides a more accurate prediction for the day-ahead electricity market and can significantly reduce resulting CO_2_ emissions through intelligent scheduling in various European countries.

The sphere of politics and legislation plays a crucial role in combating greenhouse gas emissions, as evidenced by a range of studies focusing on regulatory measures and policy implications at both international and national levels. Wang et al. [24] emphasize the urgent need for innovative technologies and systemic reforms to achieve carbon neutrality by 2050, highlighting solutions in renewable energy, food system transformation, and carbon-negative manufacturing. Their comprehensive review suggests a collaborative global effort for sustainable development and climate change mitigation. Salvia et al. [25] analyze the mitigation targets of 327 European cities, revealing that while 78 % have greenhouse gas emissions reduction targets, their average target of 47 % falls short of the Paris Agreement's goals. The study underscores the need for European cities to double their climate mitigation efforts to align with global objectives. Hsu et al. [26] investigate China's efforts towards sustainable environmental management, focusing on the role of eco-innovation, renewable energy, and environmental taxes in reducing carbon emissions. Their findings highlight the significant impact of these factors on reducing CO_2_ emissions and haze pollution, while noting that globalization tends to increase carbon emissions in the country. Nong et al. [27] conduct a comparative analysis of a global carbon tax on CO_2_ and non-CO_2_ emissions, illustrating significant deviations in economic impacts when non-CO_2_ emissions are not considered. Their study indicates higher economic contraction rates in developing countries compared to developed nations under such tax scenarios. Romero and Gramkow [28] provide insights into how economic complexity can contribute to reducing greenhouse gas emission intensity and per capita emissions. They introduce the Product Emission Intensity Index (PEII) to analyze products associated with higher emission intensities, facilitating the formulation of policies to shift production away from high-emission intensity products. Qin et al. [29] evaluate the role of environmental policy, green innovation, and a composite risk index in achieving carbon neutrality targets for G7 economies. Their results affirm the importance of strengthening environmental policies and promoting green innovation and renewable energy R&D for sustainable environmental management. Liu et al. [30] assess the effectiveness of China's Emissions Trading Scheme (ETS) in reducing PM_2.5_ levels, demonstrating that the ETS not only addresses CO_2_ emissions but also provides co-benefits by reducing air pollution. The study highlights the potential of ETS in generating positive environmental impacts beyond its primary objective. Shi et al. [31] examine the emission reduction effects of China's carbon trading pilot policy, particularly focusing on the role of carbon quota allocation and carbon trading price. Their findings suggest that the policy has effectively reduced regional carbon emissions and that the allocation method and market participation are key factors influencing emission reduction outcomes.

Integrative and holistic approaches to solving problems related to greenhouse gas emissions are gaining prominence, with a focus on comprehensive proposals and innovative solutions. Keith et al. [32] propose a comprehensive approach to carbon accounting, covering both stocks and flows, which reveals the greater mitigation benefit from protecting carbon stocks in natural forests over just focusing on carbon sequestration. This holistic approach underscores the necessity of comprehensive accounting for evaluating nature-based solutions in climate mitigation. Nilsson et al. [33] present an industrial policy framework aimed at transforming energy and emissions intensive industries towards zero emissions. They emphasize the need for profound technological and organizational changes across entire material value chains, from primary production to recycling, to achieve zero emissions. Yazdanie and Orehounig [34] address the gaps in urban energy system planning and modeling, offering solutions to improve data gaps, integrate modeling approaches, and enhance institutional frameworks. Their recommendations aim to enable urban energy planners and policymakers to make more informed and effective decisions for reducing greenhouse gas emissions. Gupta et al. [35] develop an integrated assessment framework to evaluate the environmental footprints of deep decarbonization in electricity generation. Their case study on Canada's electricity sector highlights the co-benefits of transitioning to renewable energy, including reductions in greenhouse gas emissions, water consumption, and system costs.

The research on game-theoretic modeling in regulating greenhouse gas emissions offers insightful perspectives on the complexities and solutions for environmental challenges. Halat and Hafezalkotob [36] explore the impact of carbon regulation policies on inventory decisions within a multi-stage green supply chain through a Stackelberg game approach. They assess the effects of government-implemented carbon regulations, such as caps and taxes, on supply chain costs and emissions, highlighting the critical balance between regulatory measures and supply chain efficiency. Amini and Kianfar [37] apply game theory models to green supply chain design, considering environmental impacts alongside economic factors. Their research utilizes Stackelberg, Nash, and cooperative game models to determine the optimal strategies for supply chain members, demonstrating how different game-theoretic approaches can influence profitability and sustainability outcomes. Lee [38] models market price analysis in coupled electricity and emissions trading markets, examining how power generations in oligopolistic electricity markets impact emissions trading. This study highlights the interconnectedness of electricity and emissions markets and the role of market power in shaping environmental outcomes. Garcia-Castro et al. [39] develop a robust stochastic model for a cooperative supply chain under the uncertainty of CO_2_ allowance prices. Their approach, which considers fluctuations in allowance prices from the European Union Emissions Trading System, offers insights into designing more resilient and sustainable supply chains. Christensen [40] presents a Bayesian game model for resource exploitation in hinterland regions, exploring sustainable development scenarios. This study provides a game-theoretic perspective on the management of biomass resources, emphasizing the importance of balancing intensified production with conservation efforts in rural and coastal regions.

These studies collectively illustrate the potential of game-theoretic approaches in addressing the complexities of greenhouse gas emissions regulation and the design of sustainable systems.

Despite the comprehensive exploration of greenhouse gas emissions across various dimensions, existing approaches often exhibit limitations in dynamically modeling the intricate relationship between policy mechanisms and emission behaviors in a manner that reflects the contingent, strategic interactions inherent in environmental management. This gap highlights a critical need for models that can accommodate the complexity of strategic decisions made by both regulators and emitters within a unified framework. Moreover, while the reviewed literature provides a solid foundation for understanding the impacts and strategies related to greenhouse gas emissions, there is a discernible need for innovative methodologies that can bridge the gap between theoretical modeling and practical, policy-relevant applications. This necessitates the development of integrative models that not only predict emission trends but also offer actionable insights for policy formulation and implementation, thus motivating the current study's endeavor to introduce a game-theoretic framework integrated with differential equations to better capture the dynamic interplay between emission sources and legislative responses. Through this novel approach, the present research aims to contribute to the refinement of environmental policy strategies by providing a more nuanced understanding of the potential outcomes and implications of different regulatory and emission reduction strategies.

## 2 Materials and methods

The proposed game-theoretic principle involving 'emission sources' and 'active legislation' is effectively described by a system of ordinary differential equations. This includes defining the phase coordinates and 'control' vectors of the participants in the 'game system', as these control vectors significantly influence the selected phase coordinates. Given that current legislation responds in accordance to the state of the environment, the environment itself can be considered a player in this system, striving to minimize anthropogenic impact with control actions denoted as $\theta_{1},\theta_{2},...\theta_{i}$. It is important to note that the control of the environment may have a probabilistic nature.

The 'emission source' player aims to maximize its impact on the environment and accordingly, its control actions $\mu_{1},\mu_{2},...\mu_{j}$. Therefore, the task at hand is to construct a mathematical model of the conflictual interaction between sources of atmospheric air generation and restoration (suitable for high-organization biological communities, such as mammals) and sources of atmospheric pollution with anthropogenic greenhouse gases.

The results of the modeling must provide the capability to make decisions regarding the manageability of such conflictual interactions. Currently, the principle of influencing greenhouse gas emission sources is based on emission charges according to the norms of existing legislation. The effectiveness of this influence primarily lies in the limited ability to pay for emissions (i.e., the emission source fund cannot have an infinite financial reserve for such payments). Consequently, anthropogenic sources of greenhouse gas emissions (hereafter referred to as enterprises) must optimize their emission volumes in accordance with their financial reserves oriented towards emission charges.

Based on the foregoing, let us outline the main positions for constructing a mathematical model of conflictual interaction.

1. The planetary system of human existence is a closed system.
2. Greenhouse gases are of anthropogenic origin.
3. The processes of generating atmospheric air and forming greenhouse gases are time-bound processes (as within a closed system, there cannot be 100 % atmospheric air or 100 % greenhouse gases).
4. The enterprises' payments for emissions are a limited resource and depend solely on the volumes of greenhouse gas emissions.
5. The processes of generating atmospheric air and forming greenhouse gases are non-stationary over time.

The architecture of the conflict interaction model must correspond to these stipulations. Considering these positions, the architecture of the mathematical model is a system of interconnected first-order differential equations describing the dynamics of absorption and reproduction of the studied resources, taking into account the 'payment' for 'loss' (i.e., the emission of greenhouse gas at each moment in time is equivalent to a loss at the corresponding step). Furthermore, acknowledging the probabilistic nature of environmental control actions, we emphasize the integration of stochastic elements into the model, enabling it to better account for the uncertainties and dynamics inherent in real-world environmental and legislative interactions. This approach not only extends the model's theoretical foundation but also underscores its practical value in offering nuanced insights for the development of targeted and effective environmental policies.

## 3 Results

### 3.1 Construction of the mathematical model for competitive interaction

The first stage in constructing the model is the functional representation of the mathematical model of conflict interaction:

(1) $$\left\{ \begin{array}{l} \begin{array}{l} \frac{dy_{1}\left(t\right)}{dt}=g\left(y_{1},y_{2},\theta_{1},\theta_{2}........\theta_{\nu }\;\right)\\ \frac{dy_{2}\left(t\right)}{dt}=g\left(y_{1},y_{2},\mu_{1},\mu_{2}........\mu_{\nu }\;\right) \end{array}\\ \frac{dy_{3}\left(t\right)}{dt}=g\left(y_{2},y_{3},k_{1},k_{2}........k_{\nu }\;\right) \end{array}\right.\text{,}$$

where $y_{1}\left(t\right)$ is the volume of atmospheric air suitable for the functioning of high-organization biological organisms at a specific moment in time [volume units] (hereinafter ‘oxygen’); $y_{2}\left(t\right)$ is the volume of anthropogenic greenhouse gases in the planet's atmosphere at a specific moment in time [volume units]; $y_{3}\left(t\right)$ is the payment for emissions of anthropogenic greenhouse gases at a specific moment in time [monetary units]; and $\theta_{i},\mu_{i},k_{i}$ are control parameters.

The second stage involves justifying the type of functions for generating oxygen u(t) [volume units/time] and greenhouse gases w(t) [volume units/time], considering the non-stationarity of these processes over time and logically integrating them into the system of differential equation (1).

Given that the processes of generating atmospheric air and forming greenhouse gases are time-limited, the functions u(t) and w(t) are proposed as solutions to the differential equations of dynamics with constraints:

(2) $$\frac{du\left(t\right)}{dt}+\left[a_{0}u\left(t\right)-a_{1}\right]u\left(t\right)=0$$

(3) $$\frac{dw\left(t\right)}{dt}+\left[b_{0}w\left(t\right)-b_{1}\right]w\left(t\right)=0$$

The choice of the class of differential equations in systems (2) and (3) is determined by their ability to represent the growth processes of the studied variables (functions u(t) and w(t)) to a predetermined threshold value. This property of systems (2) and (3) of differential equations demonstrates the model's ability to reach a state of equilibrium.

Specifically, for the solution of differential equations systems (2) and (3), the growth of u(t) and w(t) has a characteristic magnitude Λ, at which all components of type $\frac{d\nu \left(t\right)}{dt}$ reach zero value.

The magnitude $\Omega =\eta /\theta_{0}$ is called the threshold of the function of limited growth, towards which the values of state variables asymptotically approach at large values of the time interval. Thus, Λ is a characteristic parameter of the function of limited growth, physically defining a certain limit value that the studied variable can reach.

In this case, the threshold values of functions u(t) and w(t) will be $\Omega_{U}=a_{1}/a_{0},\Omega_{W}=b_{1}/b_{0}$ respectively.

Functions u(t) and w(t) represent the volumes of oxygen and greenhouse gases at any given time.

Note, that u(t)+w(t)=const, and $\Omega_{U}\left(t\right)+\Omega_{W}\left(t\right)=const$.

The third stage of constructing the mathematical model involves justifying the ‘payment’ for the emissions of greenhouse gases.

Since the functions for generating oxygen u(t) [volume units/time] and greenhouse gases w(t) [volume units/time] satisfy the differential equations with limited growth, the ‘payment’ for greenhouse gas emissions must also be limited. The ‘payment’ function cannot logically and practically grow infinitely over time. Hence, the ‘payment’ function must satisfy the differential equation for processes with limited dynamics over time.

Assuming that the ‘payment’ for greenhouse gas emissions is a continuous function of time, the following differential equation is proposed:

(4) $$\frac{dp\left(t\right)}{dt}-k\cdot \left[1-\frac{p\left(t\right)}{Q}\right]w\left(t\right)=0\text{,}$$

where p(t) is the payment for emissions of anthropogenic greenhouse gases at a specific moment in time [monetary units]; k is a control parameter; Q is the threshold value of ‘payment’; w(t) is the function of generating greenhouse gases [volume units/time].

The final stage of constructing the mathematical model, based on ‘game’ principles applied to the players ‘emission sources’ and ‘active legislation’ (in terms of setting payments for emissions), involves considering the factor of irregularity in the process of generating oxygen and greenhouse gases due to both natural and anthropogenic factors, as well as probabilistic factors.

In general, obtaining an analytical dependency on time for the factor of irregularity in the process of generating oxygen and greenhouse gases is a complex mathematical task requiring separate research.

One approach to solving this task is proposed through interpolation, based on experimental data obtained over a significant period. A limitation of this approach is the error of interpolation, which cannot be eliminated.

To facilitate computational experiments using the proposed mathematical model, the functions of irregularity in the process of generating oxygen and greenhouse gases over time (normalized) are presented in the following form.

- Function A(t) of irregularity in the process of generating oxygen over time:

(5) $$A\left(t\right)=\mathrm{Sin}\;{\left(0.1\cdot t\right)}^{2}+4\cdot \mathrm{Cos}\;{\left(0.2\cdot t\right)}^{2}$$

- Function B(t) of irregularity in the process of generating greenhouse gases over time:

(6) $$B\left(t\right)=\mathrm{Sin}\;{\left(0.3\cdot t\right)}^{2}$$

Thus, all stages of constructing the mathematical model based on ‘game’ principles applied to the players ‘emission sources’ and ‘active legislation’ (in terms of setting payments for emissions) are fully described (equations (4), (5), (6)), resulting in a system of three first-order linear differential equations:

(7) $$\left\{ \begin{array}{l} \begin{array}{l} \frac{dy_{1}\left(t\right)}{dt}=\varphi \cdot u\left(t\right)\cdot \left(1-\frac{y_{1}\left(t\right)}{N}\right)\cdot A\left(t\right)-\gamma \cdot y_{2}\left(t\right)+\alpha \cdot y_{2}\left(t\right)\\ \frac{dy_{2}\left(t\right)}{dt}=\beta \cdot w\left(t\right)\cdot \left(1-\frac{y_{2}\left(t\right)}{K}\right)\cdot B\left(t\right)-\theta \cdot y_{1}\left(t\right)\; \end{array}\\ \frac{dy_{3}\left(t\right)}{dt}=k\cdot \left[1-\frac{y_{3}\left(t\right)}{Q}\right]w\left(t\right) \end{array}\right.\text{,}$$

where $y_{1}\left(t\right)$ is the volume of atmospheric air suitable for the functioning of high-organization biological organisms at a specific moment in time [volume units] (hereinafter ‘oxygen’); $y_{2}\left(t\right)$ is the volume of anthropogenic greenhouse gases in the planet's atmosphere at a specific moment in time [volume units]; $y_{3}\left(t\right)$ is the payment for emissions of anthropogenic greenhouse gases at a specific moment in time [monetary units]; γ is the parameter of negative impact of greenhouse gas generation on the volume of atmospheric air suitable for high-organization biological organisms at a specific moment in time; α is the parameter of positive impact of greenhouse gases on the volume of atmospheric air through their conversion by biological organisms; θ is the parameter that inhibits the formation of greenhouse gases through the generation of oxygen; φ,β,k are exponential development parameters.

Considering (5) and (6), the system of differential equation (7) takes the form:

(8) $$\left\{ \begin{array}{l} \begin{array}{l} \frac{dy_{1}\left(t\right)}{dt}=\varphi \cdot u\left(t\right)\cdot \left(1-\frac{y_{1}\left(t\right)}{N_{U}}\right)\cdot \left(\mathrm{Sin}\;{\left(0.1\cdot t\right)}^{2}+4\cdot \mathrm{Cos}\;{\left(0.2\cdot t\right)}^{2}\right)-\gamma \cdot y_{2}\left(t\right)+\alpha \cdot y_{2}\left(t\right)\\ \frac{dy_{2}\left(t\right)}{dt}=\beta \cdot w\left(t\right)\cdot \left(1-\frac{y_{2}\left(t\right)}{K_{W}}\right)\cdot \left(\mathrm{Sin}\;{\left(0.3\cdot t\right)}^{2}\right)-\theta \cdot y_{1}\left(t\right)\; \end{array}\\ \frac{dy_{3}\left(t\right)}{dt}=k\cdot \left[1-\frac{y_{3}\left(t\right)}{Q}\right]w\left(t\right) \end{array}\right.$$

Considering the solution of differential equations (2), (3), the final version of the model (8) is obtained as:

(9) $$\left\{ \begin{array}{l} \begin{array}{l} \frac{dy_{1}\left(t\right)}{dt}=\varphi \cdot \left(\frac{U_{0}\cdot N\cdot e^{rt}}{N-U_{0}+U_{0}\cdot e^{rt}}\right)\cdot \left(1-\frac{y_{1}\left(t\right)}{N_{U}}\right)\cdot \left(\mathrm{Sin}\;{\left(0.1\cdot t\right)}^{2}+4\cdot \mathrm{Cos}\;{\left(0.2\cdot t\right)}^{2}\right)-\gamma \cdot y_{2}\left(t\right)+\alpha \cdot y_{2}\left(t\right)\\ \frac{dy_{2}\left(t\right)}{dt}=\beta \cdot \left(\frac{W_{0}\cdot K\cdot e^{\mu t}}{K-W_{0}+W_{0}\cdot e^{\mu t}}\right)\cdot \left(1-\frac{y_{2}\left(t\right)}{K_{W}}\right)\cdot \left(\mathrm{Sin}\;{\left(0.3\cdot t\right)}^{2}\right)-\theta \cdot y_{1}\left(t\right)\; \end{array}\\ \frac{dy_{3}\left(t\right)}{dt}=k\cdot \left[1-\frac{y_{3}\left(t\right)}{Q}\right]w\left(t\right) \end{array}\right.$$

### 3.2 Experimental part

Considering the significant complexity of the system of differential equation (9), its solution is proposed using numerical methods. System (9) is solved using the Runge-Kutta method with a fixed step in the Python or MathCAD environment.

The control parameter values are listed in Table 1.

**Table 1:** Control parameter values.

Control Parameter	Value	Dimension
φ	0.0088	–
β	0.2	–
k	0.00388	[1/time]
θ	0.007	[1/time]
γ	0.244	[1/time]
α	0.3	[1/time]
μ	0.91	–
r	0.82	–

Source: Authors own work.

**Fig. 1:**
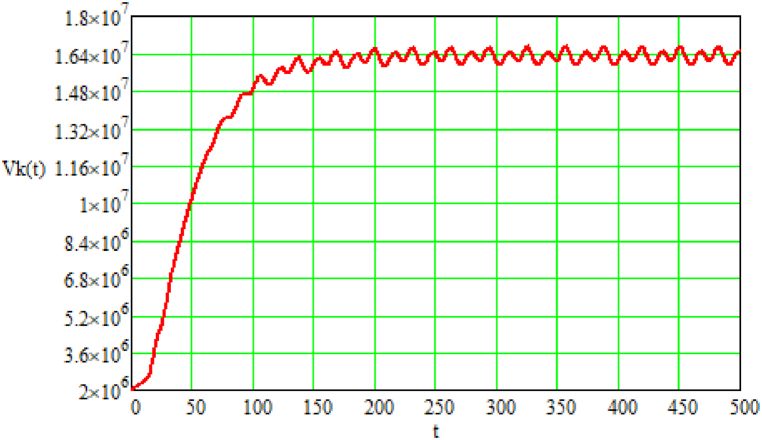
Results of modeling the volume (Vk(t)) of atmospheric air suitable for the functioning of high-organization biological organisms at a specific moment in time [volume units]; t – time (provided in conditional units depending on the observation period). Source: Authors own work.

**Fig. 2:**
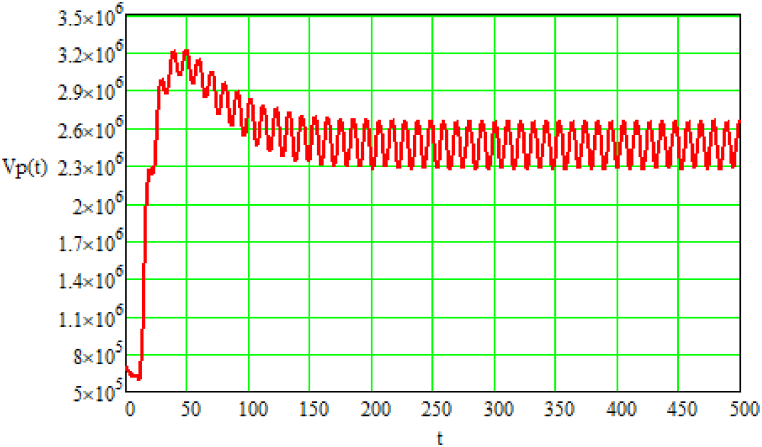
Results of modeling the volume (Vp(t)) of greenhouse gases at a specific moment in time [volume units]; t – time (provided in conditional units depending on the observation period). Source: Authors own work.

**Fig. 3:**
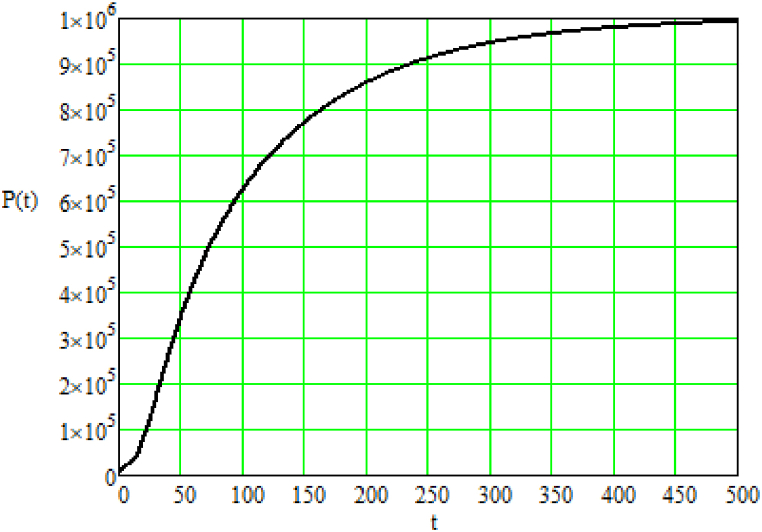
Payment P(t) for emissions of anthropogenic greenhouse gases at a specific moment in time [monetary units]; t – time (provided in conditional units depending on the observation period). Source: Authors own work.

Graphically, the results of modeling the volume of atmospheric air suitable for the functioning of high-organization biological organisms at a specific moment in time [volume units], the volume of anthropogenic greenhouse gases in the planet's atmosphere at a specific moment in time [volume units], and the payment for emissions of anthropogenic greenhouse gases at a specific moment in time [monetary units] are represented in Fig. 1, Fig. 2, Fig. 3, respectively.

From the graph in Fig. 1, a clear saturation zone is observed, as well as fluctuations relative to the saturation boundary, which are caused by the irregularity of the oxygen generation process. The threshold value of the saturation zone directly depends on the intensity of technological processes, the effectiveness of preventive measures, and natural factors (green plantations, etc.) that reduce the volumes of greenhouse gas emissions into the planet's atmosphere.

### 3.3 Innovations and interpretation enhancements

Our model introduces experimental functions to capture the dynamic and irregular nature of greenhouse gas emissions, a feature that sets it apart from existing models. This advancement not only increases the realism of our simulations but also broadens the model's applicability in crafting and evaluating environmental policies. Furthermore, recognizing the probabilistic nature of environmental control actions, our methodology incorporates stochastic elements. This addition enriches the model's ability to simulate the complex interactions between emission sources and regulatory frameworks, reflecting the uncertainties inherent in environmental management. The model's comprehensive architecture, detailed through a system of differential equations, now thoroughly accounts for the irregularities in generating oxygen and greenhouse gases. This adjustment allows for a more accurate depiction of atmospheric processes, addressing both natural variations and anthropogenic impacts. To address the complexity of our model and the necessity for robust solution techniques, we have employed the Runge-Kutta method, a numerical approach that enhances the precision of our results. This methodological choice underscores our commitment to delivering scientifically rigorous and actionable insights. The graphical representation of our results, particularly the identification of saturation zones and the illustration of fluctuations due to irregular generation processes, underscores the practical value of our model. These findings not only validate our theoretical approach but also offer a solid foundation for developing targeted strategies to reduce greenhouse gas emissions. By integrating these enhancements, our research further solidifies its contribution to the field of environmental management. Our model stands as a novel and valuable tool for policymakers and environmental scientists, providing a sophisticated mechanism for strategic planning and decision-making in the pursuit of sustainable environmental outcomes.

## 4 Discussion and future research

The modeling results of the volume (Vp(t)) of greenhouse gases (illustrated in Fig. 2) indicate a dynamic development process at the initial stage; this is followed by a fluctuating process of decreasing the volume of greenhouse gases to a certain threshold value, influenced by the generation of oxygen. Beginning at a conditional time point t = 150 [time units], the process fluctuates around this threshold value. These oscillations occur due to the unevenness in the formation of greenhouse gases.

From the modeling results, it can be concluded that the processes of generating atmospheric air suitable for the functioning of high-organization biological organisms and greenhouse gases gradually approach their threshold values over time, despite the irregularities in their formation. Accordingly, the threshold values are dependent on the intensity of technogenic load and the efficiency indicators of oxygen generation and suppression of greenhouse gas formation. However, the irregularity in the formation of atmospheric air and greenhouse gases does not lead to a decrease or increase in the corresponding threshold values of saturation zones, but only affects the level of inertia in the dynamics of generation processes.

Regarding the payment for emissions of anthropogenic greenhouse gases into the atmosphere, it can be concluded that enterprises should at least maintain a stable level of greenhouse gas emissions. It is also advisable to shorten the implementation period of effective systems for neutralizing or reducing greenhouse gas emissions, as the graph in Fig. 3 shows a significant level of payment at the time when the processes of oxygen and greenhouse gas generation are within their threshold values.

The observed dynamic development process of greenhouse gas volumes and the subsequent oscillations around threshold values underscore the critical balance between anthropogenic activities and natural regulatory mechanisms. These results highlight the necessity for advanced strategies in managing emissions, advocating for a more agile and responsive approach in the deployment of emission reduction technologies. Our work seeks to pave the way for innovative solutions that can adeptly navigate the complexities of greenhouse gas emissions regulation, ultimately contributing to the global effort towards environmental sustainability.

Future research is planned to investigate the factor of irregularity in the process of generating oxygen and greenhouse gases, influenced both by natural and anthropogenic factors, as well as by probabilistic factors. Additionally, it is necessary to define a set of control vectors that ensure an effective level of functioning of the studied 'game' system. For accomplishing these tasks, the use of artificial intelligence systems is planned. However, it should be noted that while artificial intelligence has the potential to significantly improve environmental management, it also poses several challenges, including: 1) incompleteness, inaccuracy, or bias in the data used for AI training; 2) the inability to account for long-term, indirect, or unforeseen consequences of ecological decisions; 3) AI decisions may prioritize economic interests, public health, or biodiversity conservation differently.

## 5 Conclusions

This study offers a groundbreaking approach to understanding and managing the complex dynamics of greenhouse gas emissions in the context of environmental policy and regulation. By integrating game-theoretic principles with advanced mathematical modeling, the research provides a nuanced perspective on the interaction between anthropogenic emission sources and regulatory frameworks. This novel approach, which incorporates components of greenhouse gas generation and free oxygen, alongside a differential equation representing payment functions, marks a significant advancement over existing models.

The findings demonstrate that the relationship between emission sources and legislative actions can be effectively conceptualized as a game-theoretic scenario. In this model, emission sources must navigate within established legal boundaries to minimize penalties, while legislative entities aim to regulate and mitigate environmental impacts. This conceptual framework allows for the exploration of optimal strategies that balance the economic and ecological implications of emissions. The model's innovation lies in its potential to guide the establishment of global quotas and unified taxation policies for greenhouse gas emissions, reflecting a trend toward global policy harmonization.

A key aspect of this research is the development and implementation of a comprehensive system of ordinary differential equations. These equations model the temporal dynamics of atmospheric gas generation and greenhouse gas emissions, acknowledging the time-bound nature of these processes within a closed planetary system. The study's approach innovatively accounts for the non-stationarity of these processes, offering a more accurate representation of real-world environmental changes. The inclusion of an experimental function for the irregularity of greenhouse gas emissions provides a realistic scenario for the operation of emission sources, demonstrating the model's adequacy.

One of the significant contributions of this research is the formulation of a fee-based regulatory mechanism. This mechanism is designed to incentivize emission reduction while recognizing the financial constraints and limitations of emission sources. The model predicts that enterprises should aim to maintain a stable level of emissions, emphasizing the importance of implementing effective emission reduction systems. This approach suggests the need for a global taxation policy on emissions, which could be particularly impactful for countries with advanced industries.

Furthermore, the research highlights the potential role of artificial intelligence (AI) in enhancing environmental management. AI systems could be instrumental in processing complex environmental data, offering predictive insights, and aiding in decision-making processes. However, the study also cautions about the challenges associated with AI, such as data incompleteness, inaccuracies, and the inability to fully predict long-term ecological consequences. This highlights the interdisciplinary nature of the approach, combining game theory, mathematical modeling, and environmental science to address complex environmental issues.

The study concludes that effective environmental management and policy-making require a multi-faceted approach. The proposed model serves as a valuable tool for policymakers and environmental scientists, providing insights for strategic planning over a 10-year horizon. It underscores the importance of considering technological efficacy in emission control and the need for constant adaptation to evolving environmental challenges. The research's application of a game-theoretic framework integrated with differential equations provides a fresh perspective on regulating greenhouse gas emissions, offering sustainable solutions to one of the most pressing global challenges.

In summary, this research offers a novel and practical framework for understanding and managing greenhouse gas emissions, providing a strategic pathway for balancing economic development with environmental sustainability. It stands as a testament to the power of interdisciplinary approaches in tackling global environmental issues, offering a blueprint for future research and policy development in the field of environmental science and management.

## Data availability statement

Data will not be required for this article.

## CRediT authorship contribution statement

**Oleksandr Maevsky:** Writing – original draft, Visualization, Validation, Supervision, Software, Resources, Methodology, Formal analysis, Conceptualization. **Maya Kovalchuk:** Writing – original draft, Visualization, Validation, Formal analysis. **Yuri Brodsky:** Writing – original draft, Visualization, Methodology. **Valentyna Stanytsina:** Writing – original draft, Methodology, Funding acquisition, Formal analysis. **Volodymyr Artemchuk:** Writing – review & editing, Writing – original draft, Validation, Supervision, Project administration, Methodology, Funding acquisition, Conceptualization.

## Declaration of generative AI and AI-assisted technologies in the writing process

During the preparation of this work, the author(s) used OpenAI's language model, ChatGPT, to verify the text and check for errors. After using this tool, the author(s) reviewed and edited the content as needed and take(s) full responsibility for the content of the publication.

## Declaration of competing interest

The authors declare that they have no known competing financial interests or personal relationships that could have appeared to influence the work reported in this paper.
